# Endothelin receptor antagonists target EDNRB and modulate the progression of idiopathic pulmonary fibrosis via anoikis-related genes

**DOI:** 10.3389/fmed.2025.1593376

**Published:** 2025-06-16

**Authors:** Pengfei Ning, Huiru Zhao, Yujie Weng, Yanhua Guo, Rong Jia

**Affiliations:** ^1^College of Computer and Information, Inner Mongolia Medical University, Hohhot, Inner Mongolia, China; ^2^Inner Monglia Maternal and Child Care Hospital, Hohhot, Inner Mongolia, China

**Keywords:** idiopathic pulmonary fibrosis, anoikis-related genes, EDNRB, endothelin receptor antagonists, biomarkers

## Abstract

Idiopathic pulmonary fibrosis (IPF) progression involves dysregulation of anoikis-related mechanisms, though the precise molecular drivers remain unclear. Through integrated analysis of IPF and normal lung tissue datasets, we identified 19 anoikis-related genes (ARGs) with EDNRB, MMP7, and CXCL12 showing significant differential expression (*p* < 0.05). Functional characterization revealed these ARGs predominantly regulate cell chemotaxis and inflammatory pathways, with protein network analysis identifying CXCL12 and CCL5 as central regulators. Clinically relevant findings demonstrated that EDNRB downregulation correlates with fibrotic progression, while ROC analysis validated multiple ARGs as diagnostic biomarkers (AUC > 0.8). Crucially, we discovered that FDA-approved endothelin receptor antagonists (bosentan/sitaxentan) attenuate fibrosis through EDNRB upregulation, positioning these repurposable drugs as novel therapeutic candidates. These findings establish EDNRB-mediated anoikis regulation as a key mechanism in IPF and urgently warrant clinical trials to validate endothelin receptor antagonists for targeted anti-fibrotic therapy.

##  Introduction

Idiopathic Pulmonary Fibrosis (IPF) is a serious and progressive lung disease that causes diffuse fibrosis in the alveoli, leading to a gradual decline in respiratory function (1). Although the exact cause of the disease remains unclear, studies suggest that environmental factors, genetic susceptibility, and cell-to-cell interactions may play significant roles in its pathogenesis. For instance, long-term exposure to pollutants like dust and smoke is a major risk factor for developing IPF (2). Furthermore, genetic factors, including mutations in telomerase genes (3) and polymorphisms in the MUC5B gene (4), are closely linked to the onset of IPF. Clinically, IPF is mainly characterized by dyspnea and a dry cough. The prognosis for patients is poor, with survival often lasting only 3–5 years after diagnosis (5).

Therefore, a deeper understanding of its pathogenesis and the search for effective treatment options have become important areas of research.

In recent years, there have been some advances in the treatment of IPF, such as the clinical application of antifibrotic drugs Pirfenidone and Nintedanib, which can slow disease progression but still cannot cure IPF completely (6, 7). Thus, further exploration of the molecular mechanisms of IPF and the development of new therapeutic strategies remain important directions for current research. Anoikis is a form of programed cell death that occurs when cells detach from the appropriate extracellular matrix, disrupting integrin connections (8). In the pathological process of IPF, anoikis is considered a key regulatory mechanism of programed cell death (9). Normally, cells undergoing apoptosis can effectively clear dead cells through self-regulatory mechanisms to maintain tissue homeostasis. However, in the lung tissue of IPF patients, this clearance process is significantly hindered, leading to the accumulation of apoptotic cells. This abnormal apoptosis not only hinders normal lung tissue repair but also promotes fibrosis (10). Studies have found that dysregulation of apoptotic mechanisms may also lead to significant exacerbation of inflammatory responses, thereby creating favorable conditions for the development of fibrosis (11). A deeper understanding of apoptosis in IPF clarifies the disease’s pathogenesis and provides a theoretical basis for developing new therapeutic strategies. Interventions targeting the anoikis-related pathways may bring new hope for the treatment of IPF (12, 13).

Bioinformatics is a vital analytical tool for assessing gene expression data and identifying target genes and molecular mechanisms linked to various diseases. The advancement and widespread use of high-throughput technologies in biomedical research have made integrated bioinformatics a promising approach to exploring the pathogenesis of IPF and its therapeutic targets. By analyzing genetic data from patients with IPF, researchers can identify active genes and their pathways involved in disease progression (14, 15). Additionally, bioinformatics enables scientists to extract valuable insights from complex data, which supports the development of new therapeutic strategies. For example, by analyzing gene expression patterns associated with IPF, researchers can discover new biomarkers, thereby accelerating the development of early diagnosis and personalized treatment (16, 17). Research in this field enhances our understanding of IPF and offers clearer guidance for future drug discovery.

In this study, we used several computational tools to examine the relationship between anoikis-associated genes (ARGs) and IPF. We identified 19 IPF-associated anoikis-related genes and performed functional enrichment analysis to better understand their roles in pulmonary fibrosis. We used Receiver Operating Characteristic (ROC) curve analysis to evaluate the diagnostic and predictive value of IPF-ARGs. We also examined the relationship between IPF-ARGs and lung tumors through expression and survival analyses. Additionally, we constructed interaction networks that included transcription factors, miRNAs, and drug targets related to these genes. Our investigation of anoikis in Idiopathic Pulmonary Fibrosis improves our understanding of the disease’s pathogenesis and suggests new strategies for treatment.

## Materials and methods

### Data acquisition and differential expression gene screening

Gene expression profile data for non-idiopathic pulmonary fibrosis diseases were downloaded from the Gene Expression Omnibus (GEO) database.^1^ We used the GEOquery package (18) in R to obtain gene expression datasets GSE53845 and GSE24206 related to IPF. The GSE53845 dataset comprises 40 IPF samples and 8 normal samples (non-idiopathic pulmonary fibrosis). All samples were collected from human lung biopsies or transplants and sequenced using the GPL6480 Agilent-014850 Whole Human Genome Microarray 4 × 44K G4112F. The GSE24206 dataset contains 17 IPF samples and 6 normal (non-idiopathic pulmonary fibrosis) samples, all derived from human whole lung tissue, sequenced using the GPL570 [HG-U133_Plus_2] Affymetrix Human Genome U133 Plus 2.0 Array platform. Both datasets share the same sequencing type, sample grouping information, and species origin, while also ensuring sufficient sample sizes and high data quality.

After normalizing the samples, we used the Limma package (19) in R to screen for differentially expressed genes (DEGs) between IPF and control samples. We set filtering thresholds at | log2 fold change| > 1 and *p*-value < 0.05. Additionally, we searched the PubMed database for studies on anoikis (20, 21) and combined the findings with information from the GeneCards database. This process led us to identify a total of 907 ARGs (Complete data in Supplementary Table 1). Finally, we identified the intersection of DEGs from the disease and normal groups in the two datasets to determine the DEGs related to IPF. We then intersected these IPF DEGs with ARGs to obtain IPF-ARGs.

### Functional and pathway enrichment analysis

To analyze the biological processes involved in the pathogenesis of IPF, we performed functional enrichment analysis (22) and pathway analysis (23) on the selected target genes using the Cluster Profiler package (24) in R, with a significance threshold set at *p* < 0.05, and visualized the enrichment analysis results using the ggplot2 package (25).

Gene Set Enrichment Analysis (GSEA) was used to evaluate the distribution patterns of genes within predefined sets in the gene expression data. This method ranks genes according to their correlation with phenotypic characteristics to assess their influence (26). We retrieved the gene sets “c2.cp.kegg.v2022.1.Hs.symbols.gmt” and “c5.go.all.v2022.1.Hs.symbols.gmt” from MSigDB (27). We performed GSEA using the clusterProfiler package in R, considering *p* < 0.05 as statistically significant. Finally, we visualized the results with the ggplot2 package.

### Molecular network construction and validation

To illustrate the interactions among IPF-ARGs, we built a gene-gene interaction network with the STRING online tool^2^ and visualized it using Cytoscape software (28).

We used the NetworkAnalyst database (29) to examine how miRNAs and transcription factors (TFs) regulate the expression of IPF-ARGs at the post-transcriptional level. First, we identified miRNAs associated with differentially expressed IPF-ARGs using TarBase. At the same time, we used the ENCODE database to identify the TFs associated with IPF-ARGs. Next, we predicted the relationships between IPF-ARGs and drugs by utilizing the DrugBank database. Finally, we employed Cytoscape software to visualize the results.

We examined the expression levels of IPF-ARGs in both the disease and normal groups across the two datasets, presenting the results in box plots. We used ROC curves to assess the diagnostic and predictive value of these genes. Genes with an area under the ROC curve (AUC) greater than 0.8 were deemed to have an accurate predictive value. The AUC values with 95% confidence intervals were computed using the DeLong test, which accounts for the correlation structure between biomarkers through covariance matrix estimation. Statistical significance was defined as non-overlapping 95% CIs between compared AUC values.

### Clinical relevance validation

The GEPIA database^3^ was used to analyze the expression differences and survival between different types of tumors and normal tissues. To further explore the relationship between IPF-ARGs and lung cancer progression, we validated the expression level differences of IPF-ARGs in lung tumors and normal tissues using the GEPIA database and plotted box plots. To investigate the relationship between IPF-ARGs and overall survival (OS) of patients, we conducted Kaplan-Meier univariate survival analysis using the Kaplan-Meier Plotter database^4^ to obtain key genes related to cancer survival.

## Results

### Differential expression analysis

**FIGURE 1:**
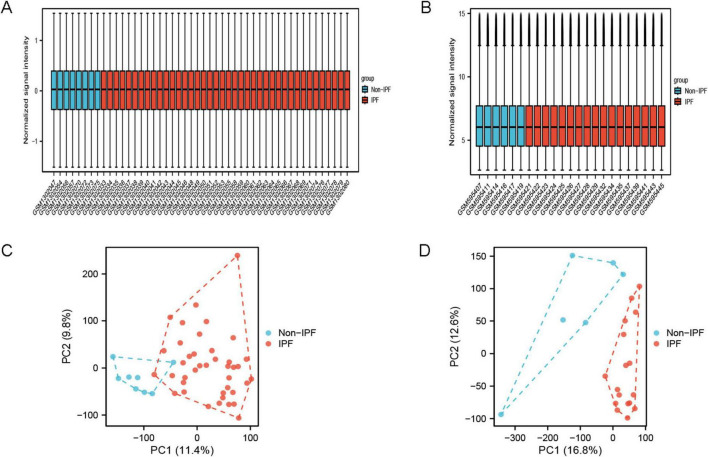
Sample normalized box plots and PCA plots. **(A)** Normalized box plot of samples from the GSE53845 dataset; **(B)** normalized box plot of samples from the GSE24206 dataset. Blue represents the normal group and red represents the disease group. **(C)** PCA plot of GSE53845 dataset; **(D)** PCA plot of GSE24206 dataset.

**FIGURE 2:**
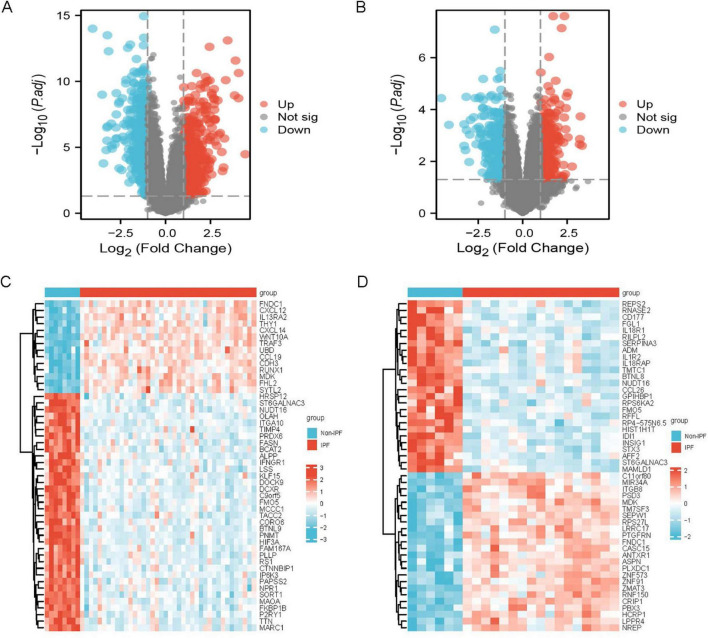
Differentially expressed genes. **(A)** Volcano plot of IPF-related DEGs in the GSE53845 dataset; **(B)** volcano plot of IPF-related DEGs in the GSE24206 dataset. **(C)** Heatmap of IPF-related DEGs in the GSE53845 dataset; **(D)** heatmap of IPF-related DEGs in the GSE24206 dataset.

**FIGURE 3:**
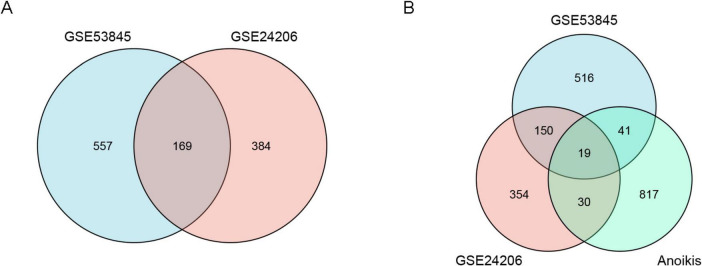
Venn diagrams of differentially expressed genes (DEGs). **(A)** The blue circle represents DEGs from the GSE53845 dataset, and the pink circle represents DEGs from the GSE24206 dataset. **(B)** The blue circle represents DEGs from the GSE53845 dataset, the pink circle represents DEGs from the GSE24206 dataset, and the green circle represents anoikis-related genes.

The sample data from datasets GSE53845 and GSE24206 were normalized, and box plots were created (Figures 1A,B). To observe the differences between samples, principal component analysis (PCA) analysis was performed, and the results were visualized using the ggplot2 package (Figures 1C,D). The differential gene analysis results showed that the GSE53845 dataset contained 726 DEGs (392 upregulated, 334 downregulated), while the GSE24206 dataset contained 553 DEGs (286 upregulated, 267 downregulated). The results were visualized using volcano plots (Figures 2A,B) and heatmaps (Figures 2C,D). The intersection of the two datasets yielded 169 differentially expressed genes related to IPF (Figure 3A). Combining the analysis of anoikis-related genes, we identified 19 IPF-ARGs: MMP7, CXCL14, PLA2G1B, TP63, THY1, CDH3, CD24, CXCL12, MAOA, MDK, CCL5, EDNRB, ITGBL1, S1PR1, FRZB, CCDC80, TGFBR3, S100A8, and TGFB2 (Figure 3B).

### Functional enrichment analysis of differentially expressed IPF-ARGs

**FIGURE 4:**
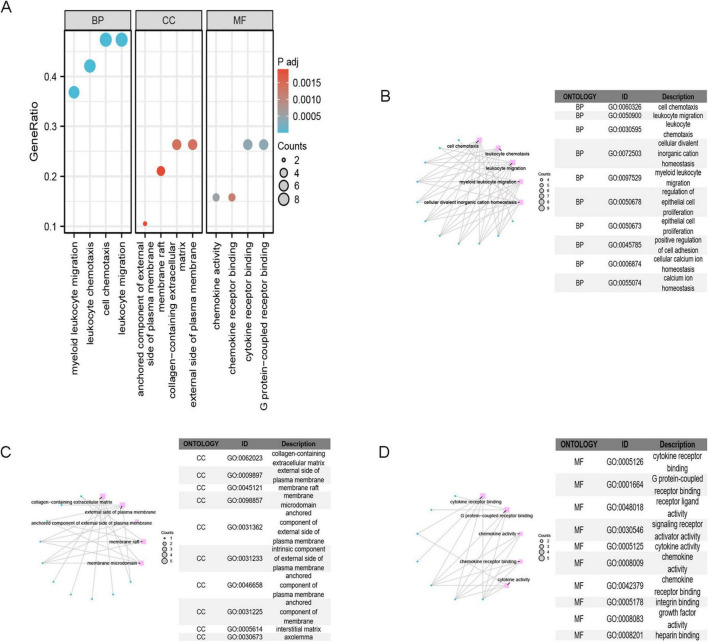
GO Enrichment Analysis. **(A)** GO enrichment analysis categorizes gene functions into three classes: Biological Process (BP), Cellular Component (CC), and Molecular Function (MF). **(B)** The top 10 terms in the BP category are displayed. **(C)** The top 10 terms in the CC category are displayed; **(D)** the top 10 terms in the MF category are displayed.

**FIGURE 5:**
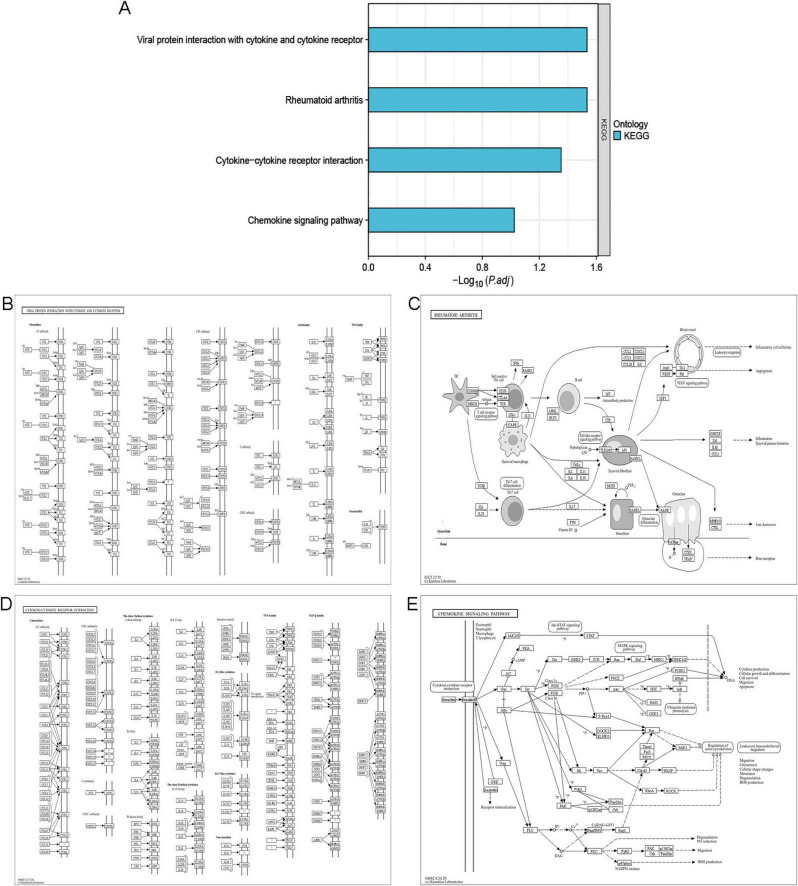
KEGG Enrichment Analysis. **(A)** KEGG enrichment analysis. **(B)** The pathway showing the interaction between viral proteins and cytokines/cytokine receptors is displayed. **(C)** The pathway of rheumatoid arthritis is displayed. **(D)** The pathway of cytokine-cytokine receptor interaction is displayed. **(E)** The pathway of chemokine signaling is displayed.

The GO enrichment analysis (Figure 4A) revealed that IPF-ARGs are mainly engaged in biological processes like cell chemotaxis, leukocyte migration, myeloid leukocyte migration, regulation of epithelial cell proliferation, positive regulation of cell adhesion, and calcium ion homeostasis, among others (Figure 4B). They are enriched in cellular components such as collagen-containing extracellular matrix, external side of plasma membrane, membrane raft, membrane microdomain, anchored component of external side of plasma membrane, intrinsic component of external side of plasma membrane, anchored component of plasma membrane, anchored component of membrane, interstitial matrix, and axolemma (Figure 4C). In terms of molecular function, GO terms related to cytokine receptor binding, G protein-coupled receptor binding, receptor ligand activity, signaling receptor activator activity, cytokine activity, chemokine activity, chemokine receptor binding, integrin binding, growth factor activity, and heparin binding were abundant (Figure 4D). The KEGG annotation of IPF-ARGs indicates that these genes are enriched in several pathways. These include viral protein interactions with cytokines and their receptors, rheumatoid arthritis, cytokine-cytokine receptor interactions, and chemokine signaling pathways (Figure 5A). These pathways emphasize the important role of cytokines and their receptors in managing immune responses, inflammation, and viral infections. Viruses may achieve immune evasion or induce immune pathological damage by interfering with these pathways, which can also become important targets for treating inflammatory diseases and viral infections (Figures 5B–E).

### Gene set enrichment analysis

**FIGURE 6:**
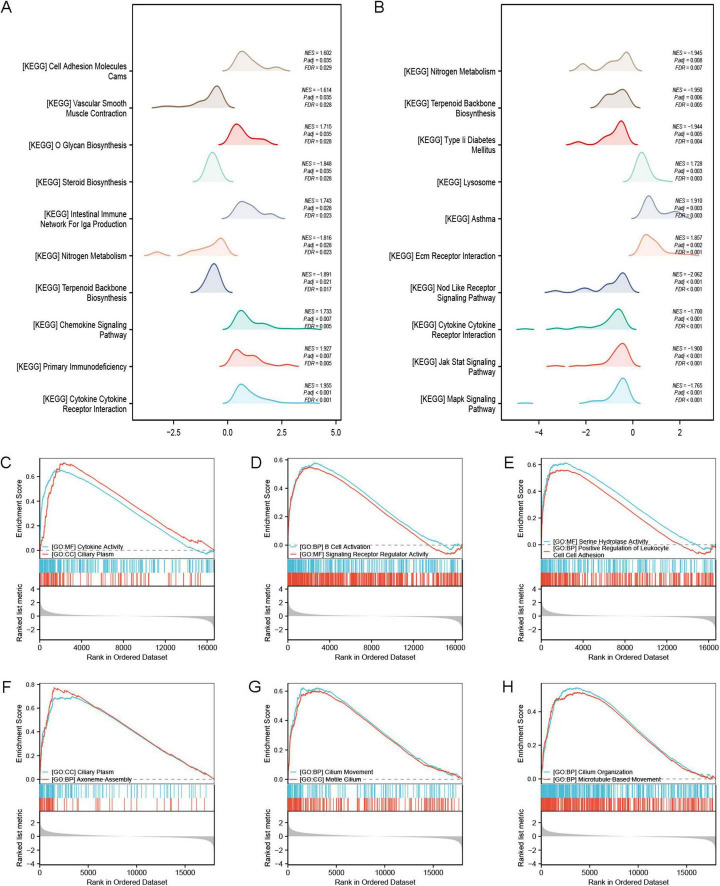
GSEA enrichment analysis. **(A)** GSEA-KEGG analysis of the GSE53845 dataset; **(B)** GSEA-KEGG analysis of the GSE24206 dataset. **(C–E)** GSEA-GO analysis of the GSE53845 dataset showing that the enriched pathways are closely related to cytokine activity, ciliary plasm, B cell activation, signaling receptor regulatory activity, serine hydrolase activity, and positive regulation of leukocyte cell-cell adhesion. **(F–H)** GSEA-GO analysis of the GSE24206 dataset showing that the enriched pathways are related to ciliary plasm, axoneme assembly, ciliary movement, motile cilium, cilium organization, and microtubule based movement.

The GSEA enrichment analysis results show that the GSE53845 dataset mainly influences pathways associated with cell adhesion molecules cams (CAMs) (NES = 1.602, FDR = 0.029), vascular smooth muscle contraction (NES = –1.614, FDR = 0.028), O glycan biosynthesis (NES = 1.715, FDR = 0.028), steroid biosynthesis (NES = –1.848, FDR = 0.028), intestinal immune network for IgA production (NES = 1.743, FDR = 0.023), nitrogen metabolism (NES = –1.816, FDR = 0.023), terpenoid backbone biosynthesis (NES = –1.891, FDR = 0.017), chemokine signaling pathway (NES = 1.733, FDR = 0.005), primary immunodeficiency (NES = 1.927, FDR = 0.005), and cytokine cytokine receptor interaction (NES = 1.955, FDR < 0.001) (Figure 6A). The GSE24206 dataset primarily impacts several key biological functions, including nitrogen metabolism (NES = –1.945, FDR = 0.007), terpenoid backbone biosynthesis (NES = –1.950, FDR = 0.005), type 2 diabetes mellitus (NES = –1.944, FDR = 0.004), lysosomal (NES = 1.728, FDR = 0.003), asthma (NES = 1.910, FDR = 0.003), extracellular matrix (Ecm) receptor interaction (NES = 1.857, FDR = 0.001), nod like receptor signaling pathway (NES = –2.062, FDR < 0.001), cytokine cytokine receptor interaction (NES = –1.700, FDR < 0.001), and both Jak stat signaling pathway (NES = –1.900, FDR < 0.001) and mark signaling pathway (NES = –1.765, FDR < 0.001) (Figure 6B).

Our findings reveal that gene expression in the two datasets influences distinct biological pathways. In the GSE53845 dataset, the genes primarily affect pathways such as cytokine activity, ciliary plasma, B cell activation, signaling receptor regulation, serine hydrolase activity, and positive regulation of leukocyte adhesion (Figures 6C–E). Genes in the GSE24206 dataset primarily control biological pathways related to ciliary plasma, axoneme assembly, ciliary movement, motile cilia, cilium organization, and microtubule-based movement (Figures 6F–H).

### Construction of protein-protein interaction networks

**FIGURE 7:**
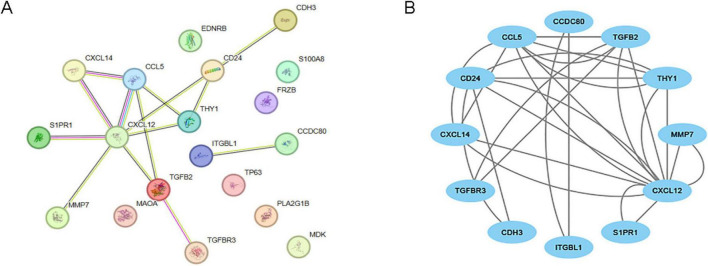
Protein-protein interaction (PPI) network. **(A)** PPI network of IPF-associated anoikis-related genes (IPF-ARGs) constructed using the STRING database. **(B)** PPI network visualization of protein interactions using Cytoscape.

Using the STRING database, we constructed a gene interaction network for IPF-ARGs, which includes 12 nodes and 28 edges. The four nodes with the strongest interactions are CXCL12, CCL5, TGFB2, and CD24 (Figure 7A). After constructing the network, we visualized it using Cytoscape software (Figure 7B).

### Network analysis of IPF-ARGs with related miRNAs, transcription factors, and drugs

**FIGURE 8:**
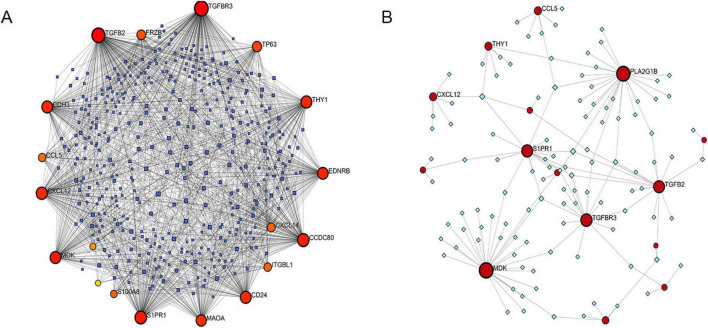
Correlation between IPF-associated anoikis-related genes (IPF-ARGs) and miRNAs and Transcription Factors (TF). **(A)** IPF-ARG-miRNA network. Blue nodes represent miRNA, and red nodes represent IPF-ARGs. **(B)** IPF-ARG-TF network. Green nodes represent transcription factors (TF), and red nodes represent IPF-ARGs.

**FIGURE 9:**
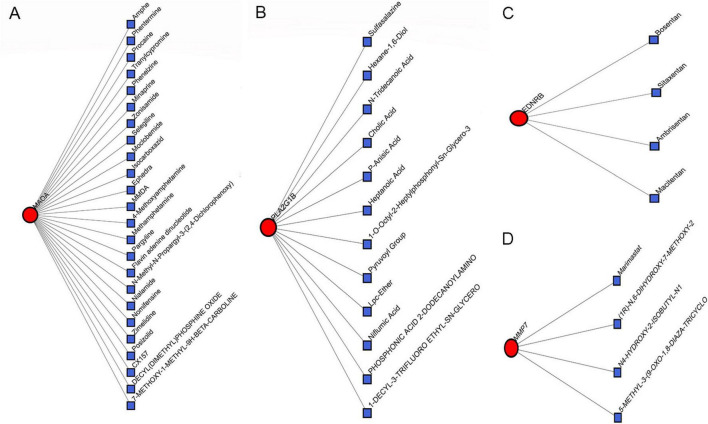
Correlation between IPF-associated anoikis-related Genes (IPF-ARGs) and Drugs. **(A–D)** The networks showing the top three drugs with the highest efficacy on IPF-ARGs are presented. Blue nodes represent drugs, and red nodes represent IPF-ARGs.

We constructed an anoikis-related gene-miRNA interaction network featuring 19 genes and 419 miRNAs linked to Idiopathic Pulmonary Fibrosis (IPF) using the NetworkAnalyst database (Figure 8A). Additionally, the IPF-related gene-transcription factor (ARG-TF) interaction network included 15 genes and 109 transcription factors (Figure 8B). The IPF-related gene-drug interaction network consists of four separate networks, with each network displaying 24, 12, 4, and 4 drug effects, respectively (Figures 9A–D).

### Expression analysis of IPF-ARGs and ROC validation

**FIGURE 10:**
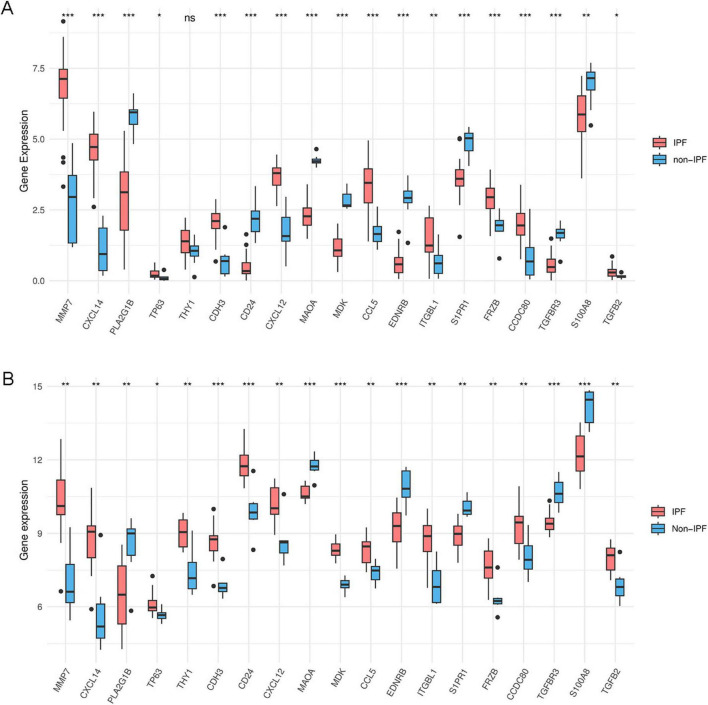
Expression of IPF-associated anoikis-related Genes (IPF-ARGs). **(A)** Expression levels of 19 IPF-ARGs in the GSE53845 dataset. Blue represents the normal group, and red represents the disease group. The abscissa indicates the IPF-ARGs, and the ordinate indicates the gene expression values (*p* > 0.05). **(B)** Expression levels of 19 IPF-ARGs in the GSE24206 dataset. Blue represents the normal group, and red represents the disease group. The abscissa represents the IPF-ARGs, and the ordinate represents the gene expression values (*p* < 0.05; *p < 0.05; **p < 0.01; ***p < 0.001; ns, not significant).

We used the ggpubr and reshape2 packages in R to create box plots that display the expression levels of IPF-ARGs in the GSE53845 and GSE24206 datasets. The analysis revealed significant differences in the expression levels of genes, including MMP7, CXCL14, PLA2G1B, TP63, CDH3, CD24, CXCL12, MAOA, MDK, CCL5, EDNRB, ITGBL1, S1PR1, FRZB, CCDC80, TGFBR3, S100A8, and TGFB2, within the GSE53845 dataset (*P* < 0.05) (Figure 10A). Similarly, the expression levels of IPF-ARGs also showed significant differences in the GSE24206 dataset (*P* < 0.05) (Figure 10B).

**FIGURE 11:**
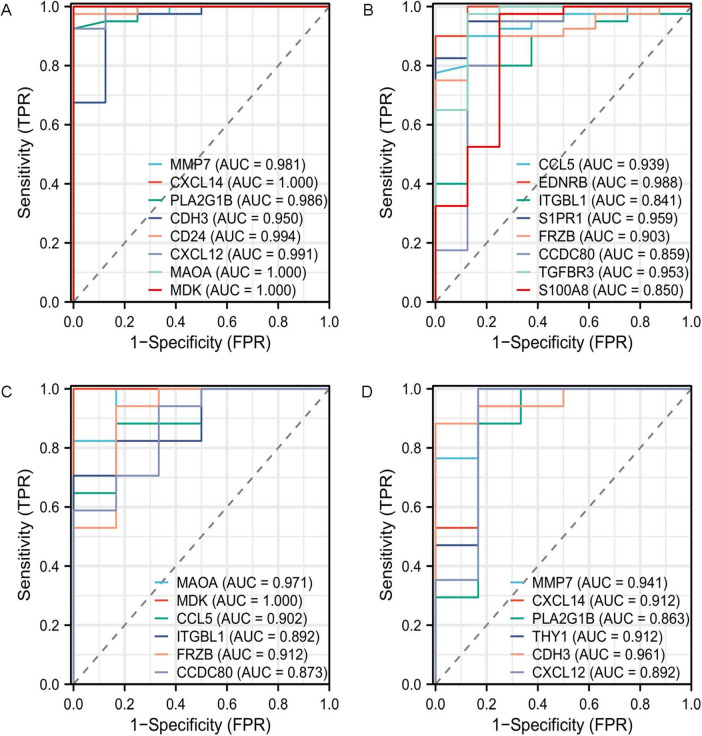
Receiver operating characteristic (ROC) curve prediction of IPF-associated anoikis-related Genes (IPF-ARGs). **(A,B)** ROC curves for IPF-associated anoikis-related genes (IPF-ARGs) in the GSE53845 dataset (AUC > 0.8). **(C,D)** ROC curves of IPF-ARGs in the GSE24206 dataset (AUC > 0.8).

ROC curve analysis revealed that multiple genes exhibited outstanding diagnostic performance across both datasets. In the GSE53845 dataset, CD24 (AUC = 0.994; 95% CI: 0.979–1.000) and EDNRB (AUC = 0.988; 95% CI: 0.960–1.000) showed the most prominent performance (Figures 11A,B), while in the GSE24206 cohort, MDK (AUC = 1.000; 95% CI: 1.000–1.000) and MAOA (AUC = 0.971; 95% CI: 0.905–1.000) demonstrated the highest discriminative power (Figures 11C,D; complete data in Supplementary Table 2).

### Expression and survival analysis of IPF-ARGs in lung cancer

**FIGURE 12:**
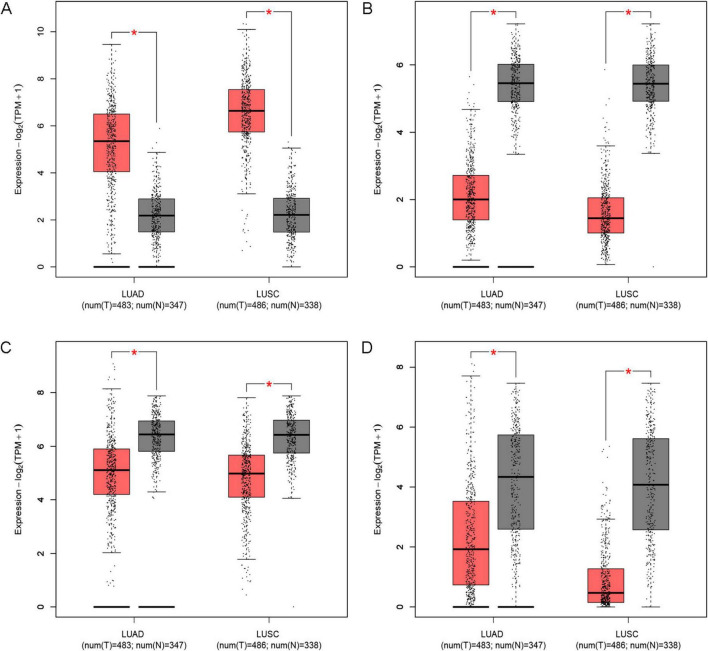
Expression of key genes in LUAD and LUSC. **(A)** CDH3; **(B)** EDNRB; **(C)** MAOA; **(D)** PLA2G1B. *Indicates a significance threshold of *p* < 0.05.

We investigated the relationship between IPF-ARGs and lung cancer progression using the GEPIA database. Our expression analysis revealed that CDH3, EDNRB, MAOA, and PLA2G1B matched our differential gene analysis results in IPF, Lung Adenocarcinoma (LUAD), and Lung Squamous Cell Carcinoma (LUSC) tissues. Notably, CDH3 was significantly upregulated, while EDNRB, MAOA, and PLA2G1B were significantly downregulated in these tissues (Figure 12).

**FIGURE 13:**
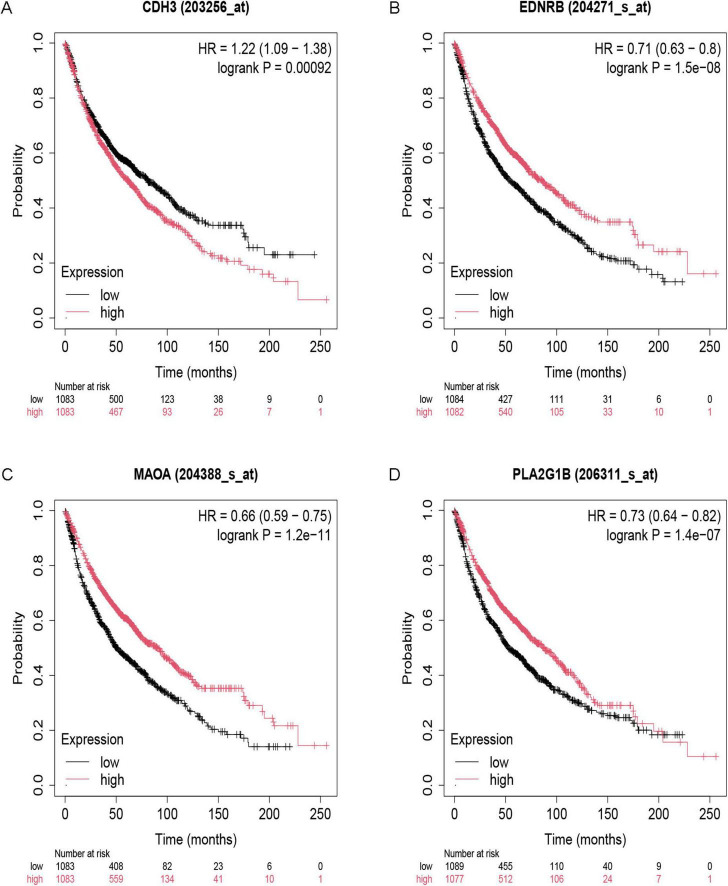
Survival analysis of key genes. **(A)** CDH3; **(B)** EDNRB; **(C)** MAOA; **(D)** PLA2G1B.

Survival analysis showed that CDH3, EDNRB, MAOA, and PLA2G1B are linked to the overall survival (OS) of patients (Figure 13). When we analyze the expression data, we observe that low expression of CDH3 is linked to longer survival; conversely, high expression of EDNRB, MAOA, and PLA2G1B is linked to longer survival. This finding indicates that CDH3 acts as an oncogene in LUAD and LUSC, while EDNRB, MAOA, and PLA2G1B serve as tumor suppressor genes in these types of lung cancer.

**FIGURE 14:**
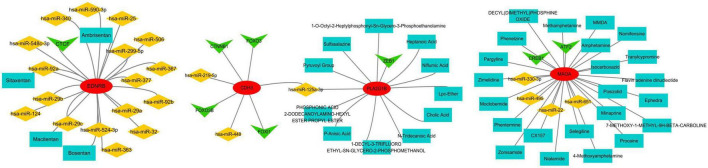
miRNA, TF, and drug target network of key genes. Red represents mRNA, green represents transcription factors (TF), yellow represents miRNA, and blue represents drugs.

We integrated the results from the prior establishment of the IPF-ARG transcription factor and miRNA networks, along with the drug target analysis of CDH3, EDNRB, MAOA, and PLA2G1B. This integration allowed us to obtain a relationship network of target genes, transcription factors, miRNAs, and drug targets (PF-ARGs-TF-miRNA-drug), which we visualized using Cytoscape (Figure 14).

The analysis of the IPF-ARGs-TF-miRNA-drug network highlighted three endothelin receptor antagonists targeting EDNRB: Bosentan, Sitaxentan, and Ambrisentan. Clinical studies suggest that endothelin receptor antagonists can improve lung function, delay the progression of idiopathic pulmonary fibrosis (IPF), and enhance patients’ quality of life (30–32). Experimental results show that Bosentan, Sitaxentan, and Ambrisentan inhibit the level of pulmonary fibrosis by upregulating EDNRB.

## Discussion

This study utilized bioinformatics analysis to explore the mechanisms of anoikis-associated genes in Idiopathic Pulmonary Fibrosis (IPF) and their potential molecular targets, focusing on the endothelin B receptor (EDNRB) and the possible use of endothelin receptor antagonists in treating IPF. We conducted differential expression analysis on two independent IPF gene expression datasets (GSE53845 and GSE24206) from the GEO database, identifying 19 genes significantly linked to anoikis in IPF. Consequently, these genes likely play significant roles in the pathogenesis of IPF.

Functional enrichment analysis indicated that these IPF-ARGs are primarily involved in biological processes such as cell chemotaxis, cell adhesion, and cytokine activity, and are enriched in pathways such as cytokine-cytokine receptor interaction and chemokine signaling pathways. Gene Set Enrichment Analysis (GSEA) also highlighted the potential roles of these genes in processes like cell adhesion and cytokine signaling. These findings indicate that anoikis dysregulation in IPF causes the accumulation of apoptotic cells, which hinders normal lung tissue repair and worsens fibrosis. Therefore, interventions targeting anoikis dysregulation may offer new insights for treating IPF.

We constructed protein-protein interaction (PPI) networks and identified genes like CXCL12, CCL5, TGFB2, and CD24 that exhibit high connectivity within the IPF-ARG interaction network, indicating their potential roles in the pathological process of IPF. Furthermore, analyzing the interaction networks of IPF-ARGs with miRNAs, transcription factors, and drugs revealed potential targets for post-transcriptional regulation and drug intervention, thereby enhancing our understanding of therapeutic strategies.

The diagnostic performance of identified biomarkers was assessed through internal validation on the original cohorts. While the observed high AUC values (e.g., EDNRB (AUC = 0.988; 95% CI: 0.960–1.000) suggest strong discriminative capacity, further external validation using prospective patient cohorts is warranted to confirm clinical applicability. Furthermore, our study investigated the relationship between IPF-ARGs and lung cancer, identifying CDH3 as an oncogenic gene and recognizing EDNRB, MAOA, and PLA2G1B as tumor suppressor genes. This finding suggests a possible link between IPF and lung cancer development. It calls for further investigation into the mechanisms that connect these two conditions.

In our study, EDNRB was confirmed as a key ARG closely related to the progression of IPF. The EDNRB (endothelin B receptor) gene is located on human chromosome 13q22.1 and encodes the endothelin B receptor (EDNRB) protein, which is part of the G protein-coupled receptor family. These receptors exhibit a high degree of conservation across species and play significant roles in various biological processes (33–35). Known biological functions of EDNRB include regulating cell proliferation, migration, and metabolic processes, with particular relevance to its anti-tumor characteristics in cancer biology. For example, decreased expression of EDNRB in prostate cancer and triple-negative breast cancer is associated with tumor progression and poor prognosis (36–38). Although specific literature on the role of endothelin receptor type B in pulmonary fibrosis is lacking, our study shows that this receptor is important for apoptosis. In the pathological process of pulmonary fibrosis, EDNRB may influence the proliferation of fibroblasts and their transition into myofibroblasts. This change is crucial for the development of lung tissue fibrosis.

Endothelin receptor antagonists (ERAs) targeting the endothelin B receptor (EDNRB) exhibit multidimensional therapeutic potential, particularly in idiopathic pulmonary fibrosis (IPF). Classified by receptor selectivity-including ETA-selective antagonists (e.g., atrasentan), ETB-selective antagonists (e.g., BQ-788), and dual ETA/ETB antagonists (e.g., bosentan)-these agents modulate distinct pathological pathways. In IPF, ETA antagonists reduce collagen deposition by 40% through TGF-β signaling inhibition, while ETB activation enhances endothelin clearance, as demonstrated in preclinical models (39). Beyond fibrosis, EDNRB dysregulation influences cancer progression: low EDNRB expression promotes metastasis via ERK pathway activation, whereas bosentan suppresses tumor cell migration by 62% and reverses epithelial-mesenchymal transition (38). Clinical translation, however, faces challenges. While atrasentan reduced proteinuria by 34% in diabetic nephropathy trials, its oncology applications are limited by fluid retention, potentially linked to EDNRB’s ceRNA regulatory network involving lncRNA FENDRR and miR-148a (40). Emerging combination strategies address these limitations-dual ET-1/PDGF pathway inhibition with nintedanib reduces IPF lung function decline by 58% annually, and ginsenoside Rg3 synergizes with PD-1 inhibitors to enhance antitumor immunity via EDNRB upregulation, achieving 2.1-fold survival improvement in murine models (41). Future efforts must prioritize EDNRB molecular profiling to balance efficacy and safety, advancing personalized ERA-based regimens for fibrotic and neoplastic diseases.

This study has limitations to acknowledge: The retrospective design of public database analysis may introduce selection bias, while computational findings require experimental confirmation. The diagnostic model needs validation across diverse populations and disease stages. EDNRB’s mechanistic role and observed IPF-cancer connections warrant functional studies to establish causality. These considerations highlight opportunities for integrating multi-omics approaches in future translational investigations.

In summary, identifying and validating risk genes associated with IPF enhances our understanding of its pathophysiology and paves the way for future clinical advancements. Integrating these genes into clinical practice as diagnostic and prognostic tools could greatly improve patient care and personalized treatment options. Future studies should aim to clarify how these biomarkers function and their interactions with different therapeutic agents. In particular, research should focus on endothelin receptor antagonists, which have shown promising clinical benefits in related conditions.

## Data Availability

The datasets presented in this study can be found in online repositories. The names of the repository/repositories and accession number(s) can be found below: https://www.ncbi.nlm.nih.gov/, GSE53845; https://www.ncbi.nlm.nih.gov/, GSE24206.

## Footnotes

1
https://www.ncbi.nlm.nih.gov/gds

2
http://string-db.org

3
http://gepia.cancer-pku.cn/

4
http://kmplot.com/analysis/

## References

[1] Richeldi L Collard HR Jones MG. Idiopathic pulmonary fibrosis. *Lancet.* (2017) 389:1941–52. 10.1016/S0140-673630866-8 28365056

[2] Gandhi SA Min B Fazio JC Johannson KA Steinmaus C Reynolds CJ The impact of occupational exposures on the risk of idiopathic pulmonary fibrosis: A systematic review and meta-analysis. *Ann Am Thorac Soc.* (2024) 21:486–98. 10.1513/AnnalsATS.202305-402OC 38096107 PMC10913770

[3] Zhang D Newton CA. Familial pulmonary fibrosis: Genetic features and clinical implications. *Chest.* (2021) 160:1764–73. 10.1016/j.chest.2021.06.037 34186035 PMC8628177

[4] Moll M Peljto AL Kim JS Xu H Debban CL Chen X A polygenic risk score for idiopathic pulmonary fibrosis and interstitial lung abnormalities. *Am J Respir Crit Care Med.* (2023) 208:791–801. 10.1164/rccm.202212-2257OC 37523715 PMC10563194

[5] Kim JW Barrett K Loke Y Wilson AM. The effect of statin therapy on disease-related outcomes in idiopathic pulmonary fibrosis: A systematic review and meta-analysis. *Respir Med Res.* (2021) 80:100792. 10.1016/j.resmer.2020.100792 34091200

[6] Honda K Saraya T Ishii H. A real-world prognosis in idiopathic pulmonary fibrosis: A special reference to the role of antifibrotic agents for the elderly. *J Clin Med.* (2023) 12:3564. 10.3390/jcm12103564 37240670 PMC10219256

[7] Waxman AB Elia D Adir Y Humbert M Harari S. Recent advances in the management of pulmonary hypertension with interstitial lung disease. *Eur Respir Rev.* (2022) 31:210220. 10.1183/16000617.0220-2021 35831007 PMC9724812

[8] Taddei ML Giannoni E Fiaschi T Chiarugi P. Anoikis: An emerging hallmark in health and diseases. *J Pathol.* (2012) 226:380–93. 10.1002/path.3000 21953325

[9] Liao Y Yang Y Zhou G Chen L Yang Y Guo S Anoikis and SPP1 in idiopathic pulmonary fibrosis: Integrating bioinformatics, cell, and animal studies to explore prognostic biomarkers and PI3K/AKT signaling regulation. *Expert Rev Clin Immunol.* (2024) 20:679–93. 10.1080/1744666X.2024.2315218 38318669

[10] Wang Q Xie ZL Wu Q Jin ZX Yang C Feng J. Role of various imbalances centered on alveolar epithelial cell/fibroblast apoptosis imbalance in the pathogenesis of idiopathic pulmonary fibrosis. *Chin Med J.* (2021) 134:261–74. 10.1097/CM9.0000000000001288 33522725 PMC7846426

[11] Lin JH Liu CC Liu CY Hsu TW Yeh YC How CK Selenite selectively kills lung fibroblasts to treat bleomycin-induced pulmonary fibrosis. *Redox Biol.* (2024) 72:103148. 10.1016/j.redox.2024.103148 38603946 PMC11017345

[12] Shetty S Idell S. Caveolin-1-related intervention for fibrotic lung diseases. *Cells.* (2023) 12:554. 10.3390/cells12040554 36831221 PMC9953971

[13] Lu X Zhu C Gao Y Yu Z Yan Q Liu Y Design, synthesis, and evaluation of pirfenidone-NSAIDs conjugates for the treatment of idiopathic pulmonary fibrosis. *Bioorgan Chem.* (2024) 143:107018. 10.1016/j.bioorg.2023.107018 38071874

[14] Kim Y Kim Y Lim HJ Kim DK Park JH Oh CM. Integrative single-cell transcriptome analysis provides new insights into post-COVID-19 pulmonary fibrosis and potential therapeutic targets. *J Med Virol.* (2023) 95:e29201. 10.1002/jmv.29201 37966390

[15] Wang Y Chen S Chen S Jiang J. Unveiling the role of copper metabolism and STEAP2 in idiopathic pulmonary fibrosis molecular landscape. *J Cell Mol Med.* (2024) 28:e18414. 10.1111/jcmm.18414 38872435 PMC11176596

[16] Luo H Yan J Zhou X. Constructing an extracellular matrix-related prognostic model for idiopathic pulmonary fibrosis based on machine learning. *BMC Pulmon Med.* (2023) 23:397. 10.1186/s12890-023-02699-8 37858084 PMC10585847

[17] Zhu H Zhou A Zhang M Pan L Wu X Fu C Comprehensive analysis of an endoplasmic reticulum stress-related gene prediction model and immune infiltration in idiopathic pulmonary fibrosis. *Front Immunol.* (2024) 14:1305025. 10.3389/fimmu.2023.1305025 38274787 PMC10808546

[18] Davis S Meltzer PS. GEOquery: A bridge between the gene expression omnibus (GEO) and BioConductor. *Bioinformatics.* (2007) 23:1846–7. 10.1093/bioinformatics/btm254 17496320

[19] Ritchie ME Phipson B Wu D Hu Y Law CW Shi W Limma powers differential expression analyses for RNA-sequencing and microarray studies. *Nucleic Acids Res.* (2015) 43:e47. 10.1093/nar/gkv007 25605792 PMC4402510

[20] Newland B Welzel PB Newland H Renneberg C Kolar P Tsurkan M Tackling cell transplantation anoikis: An injectable, shape memory cryogel microcarrier platform material for stem cell and neuronal cell growth. *Small.* (2015) 11:5047–53. 10.1002/smll.201500898 26237446 PMC5656175

[21] Paoli P Giannoni E Chiarugi P. Anoikis molecular pathways and its role in cancer progression. *Biochim Biophys Acta.* (2013) 1833:3481–98. 10.1016/j.bbamcr.2013.06.026 23830918

[22] Gene Ontology, Consortium. Gene ontology consortium: Going forward. *Nucleic Acids Res.* (2015) 43:D1049–56. 10.1093/nar/gku1179 25428369 PMC4383973

[23] Kanehisa M Sato Y Kawashima M. KEGG mapping tools for uncovering hidden features in biological data. *Protein Sci.* (2022) 31:47–53. 10.1002/pro.4172 34423492 PMC8740838

[24] Yu G Wang LG Han Y He QY. clusterProfiler: An R package for comparing biological themes among gene clusters. *Omics.* (2012) 16:284–7. 10.1089/omi.2011.0118 22455463 PMC3339379

[25] Wickham H. *ggplot2: Elegant graphics for data analysis.* New York, NY: Springer-Verlag (2016).

[26] Subramanian A Tamayo P Mootha VK Mukherjee S Ebert BL Gillette MA Gene set enrichment analysis: A knowledge-based approach for interpreting genome-wide expression profiles. *Proc Natl Acad Sci USA.* (2005) 102:15545–50. 10.1073/pnas.0506580102 16199517 PMC1239896

[27] Liberzon A Birger C Thorvaldsdóttir H Ghandi M Mesirov JP Tamayo P. The molecular signatures database (MSigDB) hallmark gene set collection. *Cell Syst.* (2015) 1:417–25. 10.1016/j.cels.2015.12.004 26771021 PMC4707969

[28] Shannon P Markiel A Ozier O Baliga NS Wang JT Ramage D Cytoscape: A software environment for integrated models of biomolecular interaction networks. *Genome Res.* (2003) 13:2498–504. 10.1101/gr.1239303 14597658 PMC403769

[29] Zhou G Soufan O Ewald J Hancock REW Basu N Xia J. NetworkAnalyst 3.0: A visual analytics platform for comprehensive gene expression profiling and meta-analysis. *Nucleic Acids Res.* (2019) 47:W234–41. 10.1093/nar/gkz240 30931480 PMC6602507

[30] Amann U Nadine Wentzell, Kollhorst B Haug U. Prescribing of endothelin receptor antagonists and riociguat in women of childbearing age in a large German claims database study. *Reprod Toxicol.* (2023) 119:108415. 10.1016/j.reprotox.2023.108415 37245698

[31] O’Shea O Murphy G Forde L O’Reilly KMA. A qualitative exploration of people living with idiopathic pulmonary fibrosis experience of a virtual pulmonary rehabilitation programme. *BMC Pulmon Med.* (2022) 22:448. 10.1186/s12890-022-02221-6 36443780 PMC9702935

[32] Nemeth J Schundner A Frick M. Insights into development and progression of idiopathic pulmonary fibrosis from single cell RNA studies. *Front Med.* (2020) 7:611728. 10.3389/fmed.2020.611728 33392232 PMC7772461

[33] Wei F Ge Y Li W Wang X Chen B. Role of endothelin receptor type B (EDNRB) in lung adenocarcinoma. *Thorac Cancer.* (2020) 11:1885–90. 10.1111/1759-7714.13474 32394530 PMC7327673

[34] Gunadi, Vujira KA Amadeus VC Gabriela GC Amaragati AR Geometri ET Aberrant expressions of EDNRB and EDN3 in a multifactorial hirschsprung disease. *Curr Pediatr Rev.* (2025). 10.2174/0115733963343518241223193627 [Epub ahead of print]. 39844409

[35] Chen Y Li H Wang J Yang S Su Z Wang W The Ednrb-Aim2-AKT axis regulates neural crest-derived melanoblast proliferation during early development. *Development.* (2024) 151:dev202444. 10.1242/dev.202444 39555938

[36] Li X Liu B Wang S Dong Q Li J. EDNRB negatively regulates glycolysis to exhibit anti-tumor functions in prostate cancer by cGMP/PKG pathway. *Mol Cell Endocrinol.* (2025) 598:112459. 10.1016/j.mce.2025.112459 39788311

[37] Liu S Zhang J Zhu J Jiao D Liu Z. Prognostic values of EDNRB in triple-negative breast cancer. *Oncol Lett.* (2020) 20:149. 10.3892/ol.2020.12012 32934717 PMC7471672

[38] Li X Liu B Wang S Dong Q Li J. EDNRB inhibits the growth and migration of prostate cancer cells by activating the cGMP-PKG pathway. *Open Med.* (2024) 19:20230875. 10.1515/med-2023-0875 38205153 PMC10775416

[39] Raghu G Remy-Jardin M Richeldi L Thomson CC Inoue Y Johkoh T Idiopathic pulmonary fibrosis (an update) and progressive pulmonary fibrosis in adults: An official ATS/ERS/JRS/ALAT clinical practice guideline. *Am J Respir Crit Care Med.* (2022) 205:e18–47. 10.1164/rccm.202202-0399ST 35486072 PMC9851481

[40] Song G Dai T Ren Y Chang Y Guo P Wang Z Understanding metabolic characteristics and molecular mechanisms of large to giant congenital melanocytic nevi: Implications for melanoma risk and therapeutic targets. *Anal Methods.* (2025) 17:3229–38. 10.1039/d5ay00122f 40190193

[41] Spagnolo P Kropski JA Jones MG Lee JS Rossi G Karampitsakos T Idiopathic pulmonary fibrosis: Disease mechanisms and drug development. *Pharmacol Ther.* (2021) 222:107798. 10.1016/j.pharmthera.2020.107798 33359599 PMC8142468

